# Evaluation of a New Algorithm‐Based Approach in Subjective Refraction

**DOI:** 10.1155/joph/5572620

**Published:** 2026-05-15

**Authors:** Imene Salah-Mabed, Vincent Péan, Damien Gatinel

**Affiliations:** ^1^ Department of Anterior Segment and Refractive Surgery, Rothschild Foundation, Paris, France

## Abstract

**Purpose:**

The aim of this study was to evaluate a new algorithm‐based approach in subjective refraction by comparing the refractions obtained by a professional expert with those obtained using the SiviewExam Expert system on a large population.

**Methods:**

Two subjective refraction methods were compared prospectively on patients visiting Rothschild Foundation between October 2022 and May 2023: (i) A manual refraction performed by an expert using a standardized method and (ii) an automated one with the SiviewExam Expert system. Outcomes of the two subjective refractions were compared using a power vector analysis method. Power vectors are a geometric representation of spherocylindrical refractive errors in 3 fundamental dioptric components: *M* (*M* = *S* + *C*/2), J0 (J0 = (−*C*/2) cos (2*α*) at an axis of *α* = 0 = 180°), and J45 (J45 = (−*C*/2) sin (2*α*) at an axis of *α* = 45°). At the end of the examination, the SiviewExam Expert system provided a report that has been analyzed as well.

**Results:**

A total of 107 patients, with a mean age of 35.7 ± 11.9 years (ranging from 19 to 69 years) were included in the study. The mean SE was −2.10 ± 3.37 D (ranging from −11 D to +7.13 D). The mean differences have shown that there were no bias between the measurements for *M* (0.01 D, −0.04–0.07 D) and J45 (0.009 D, −0.01–0.03 D) and a clinically negligible bias (0.05 D, −0.08–−0.02 D) in the estimation of J0. The limits of agreement were −0.54 D (lower limit: −0.63–−0.44) to 0.56 D (upper limit: 0.47–0.65), −0.22 D (lower limit: −0.26 to −0.18) to 0.24 D (upper limit: 0.20–0.27), –0.34 D (lower limit: –0.39 to –0.29) to 0.25 D (upper limit: 0.20 to 0.30) for *M*, J45, and J0, respectively, and were significantly lower than the expected inter‐examiner variability of 0.7 D. Finally, the report provided by the SiviewExam Expert system was found to be accurate according to the expert in 100% of the cases.

**Conclusion:**

Our outcomes show that in a large adult population of over 100 patients with a wide range of ametropias, there were no statistically significant differences in the results for *M*, J0, and J45 between the two refraction methods, indicating that we could reasonably clinically consider the SiviewExam Expert system examination comparable with the one conducted by an expert.

## 1. Introduction

Refractive disorders, such as myopia, pose a significant public health issue worldwide. The prevalence of myopia has seen a substantial increase in recent decades, currently impacting 2.6 billion individuals, with projections estimating this number could rise to 3.3 billion by 2030, including 312 million children [1, 2]. Additionally, vision disorders continue to affect millions of patients, particularly in regions with limited access to healthcare services [3].

In healthcare deserts, the lack of eye care professionals and specialized equipment deprives many people of proper vision correction, impacting their quality of life and economic productivity. According to the *World Economic Forum* [4], the global cost of uncorrected poor vision is estimated at $272 billion per year. Additionally, the quality of visual correction management varies due to differences in methodologies, professionals’ expertise, physiological factors, patient cooperation, and the time spent on subjective refractions [5]. Given the worldwide shortage of eye care professionals, particularly in medically underserved regions, implementing automated or semi‐automated subjective refraction systems will play a vital role in reducing access barriers and improving public health outcomes in the next few years.

Objective refractive solutions could then appear as the obvious answer to these problems. However, although autorefractometers provide a reference measurement on which to base subjective refraction and appear to yield good results regarding measurement repeatability [6, 7], their main limitation is an overestimation of myopia or an underestimation of hyperopia [8–10], as well as a failure to consider patient comfort in binocular vision with the correction found [11, 12]. Currently, the reference method used to determine refractive error, and considered as the gold standard, is the subjective refraction [13–15]. The main issue is that the realization of subjective refraction is time‐consuming, and the correction could fluctuate due to examiner’s or patient’s factors [16].

With the growing need to optimize patient care for better access to ophthalmological services and more accurate, cost‐effective examinations, automatic systems for evaluating patients’ subjective refraction are now essential. The rise of different solutions based on AI algorithms, teleconsultation, and tele expertise enable this [17–26]. Yet, to date, these systems have not demonstrated sufficient clinical accuracy to measure subjective refraction in healthy subjects. It is crucial to develop systems that adapt to all cases, including complex ones, while ensuring accuracy and tailored visual analysis comparable to those provided by experienced visual health experts. SiviewExam is a French‐developed Expert system designed to enhance the process of subjective refraction through algorithmic automation. The platform employs advanced computational models to interpret patient responses during visual testing, aiming to replicate the decision‐making process of an experienced practitioner. By standardizing the subjective refraction procedure, the SiviewExam Expert system aims to improve measurement repeatability and interexaminer reproducibility. At the conclusion of the examination, the Expert system generates a report summarizing the visual findings and suggesting appropriate optical corrections tailored to the patient’s responses.

The aim of this study was to evaluate this new algorithm‐based approach in subjective refraction by comparing the refractions obtained by a professional expert (using the mono/bio/bino method​ [27]) with those obtained using the SiviewExam® Expert system on a large population. The accuracy of the final report from the SiviewExam® Expert system was also assessed.

## 2. Patients and Methods

### 2.1. The Theoretical Basis of Traditional Subjective Refraction and Its Limits

Unlike objective refraction, which uses instruments such as the autorefractometer to measure refractive errors, subjective refraction relies on the patient’s responses to visual tests to determine their optimal optical correction based on their visual preferences [28].

Subjective refraction of the patients is determined using test glasses with power steps of 0.25 D. The methods currently employed have origins in the early twentieth century. Specifically, the “fog method” is derived from research published by English physicist William Swaine in 1925, concerning the correlation between defocusing and visual acuity [29]. The technique of finding the cylinder using crossed cylinders with reversal was developed by American ophthalmologist Edward Jackson in 1907. This method of refraction has remained largely unchanged; however, the instruments used for presenting glasses have evolved from trial glasses, known since the nineteenth century, to the manual refractor introduced around 1930, and later to the automatic refractor that became widespread around the 2000s. These refractors operate by presenting combinations of spherical and cylindrical glasses before the patient’s eye in steps of 0.25 D, according to the classical technique accepted universally, which includes four successive steps [27]: (1) determining the power of the sphere, (2) determining the cylinder axis, (3) determining the cylinder power, and (4) adjusting the sphere.

By design, refractors limit the accuracy of refraction to a pitch of 0.25 D due to the glasses used and the method employed. They separately adjust the sphere power, cylinder power, and cylinder axis orientation. These limitations affect examination time and measurement repeatability. Zadnik et al. report a repeatability limit exceeding +0.71 D in spherical equivalent, especially with different examiners [6, 16, 30]. A later paper used the same raw data from the same 40 subjects in the Zadnik paper to re‐evaluate the repeatability of subjective refraction. In that paper, the 95% limits of agreement (LoAs) of the spherical equivalent component of subjective refraction was reported to be ±0.51 D [31].

### 2.2. The SiviewExam Algorithm

**FIGURE 1:**
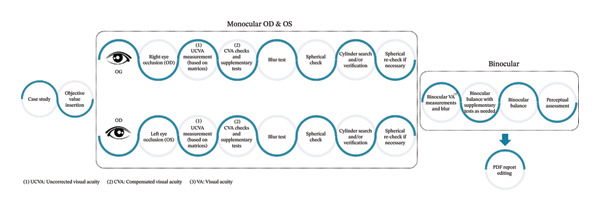
General steps of the SiviewExam expert system examination.

The SiviewExam® Expert system algorithm operates a refractive head through a tablet and follows two key principles:

1. The algorithm automatically guides the practitioner, step by step, to achieve optimal correction, mimicking the expertise of a professional. It is based on the theoretical framework of sequential tests using the mono‐bio‐bino method [27] (as shown in Figure 1). Each question issued to the patient is a closed question dictated by the system, requiring a specific response from the patient. To ensure the most accurate examination possible, the algorithm uses a database of over 500 patient profiles, factoring in criteria such as the patient’s age and degree of ametropia. Additionally, the patient’s responses throughout the process help the algorithm navigate a logical examination path.
2. The algorithm can detect abnormal responses, identifying measurement errors and differentiating between issues caused by the equipment and those arising from patient misunderstanding. These control loops enable the algorithm to detect potential malfunctions or suspect the presence of conditions like cataracts, keratoconus (KC), and others.

Beyond delivering final subjective refraction data, the SiviewExam® Expert system provides practitioners with a comprehensive summary of all the measurements taken during the examination, highlights any detected anomalies, and offers tailored optical correction suggestions. These recommendations may include ergonomic advice or visual exercises to enhance patient care.

### 2.3. Patients

This study included 107 patients visiting the Rothschild Foundation from October 2022 to May 2023. All patients underwent complete ocular examination prior to subjective refraction assed by the SiviewExam® Expert system and by an optometrist expert, including slit lamp and nondilated fundus examination. Corneal topography was performed with the Pentacam® (Bausch & Lomb®, USA).

Patients presenting with corneal disease or other ocular pathology (amblyopia, glaucoma, cataract, retinopathy, nystagmus, and strabismus), those with evidence of subclinical KC, or those with a history of recent ocular surgery were excluded from the study. We also excluded patients whose eyes tested positive for KC or keratoconus suspect (KCS), as diagnosed by the Corneal Navigator Neural Network, which utilizes Klyce and Maeda indices on the OPD‐Scan III (Nidek, Japan).

We included patients older than 18 years and able to read the Latin alphabet, with a monocular visual acuity of +0.1 LogMAR or better. All patients provided written informed consent. The study and data acquisition were achieved with approval from the independent ethical review board South Mediterranean III (France, No. 2019.09.10_cinq_19.01641.190910. Informed consent was obtained from each patient after they voiced understanding of the purpose and procedures in the study, in accordance with the Declaration of Helsinki.

### 2.4. Subjective Refraction’s Evaluation

Each patient underwent in a random order: (1) a subjective refraction assessed by the SiviewExam® Expert system and (2) one manually by the same optometrist expert (IS), in the same conditions, and both based on the same initial objective refraction measurement from the Tonoref 3 autorefractometer (Nidek, Gamagori, Japan). In addition, the SiviewExam® Expert system final report has been evaluated by the expert, by assigning a scoring of 1 if judged correct and 0 if incorrect.

The manual subjective refraction was based on the standardized protocol of the “mono‐bio‐bino” [27] following classic steps: (1) spheric component check, (2) cylinder component check, (3) second spheric component check, (4) binocular balance to achieve maximum convex sphere with maximum visual acuity, and (5) final comparison corrections to obtain the final comfortable prescription.

SiviewExam® Expert system examinations were conducted by two optometrists (MSc). The examination began systematically with an interview, aimed at gathering information about the patient. This interview is the key for the algorithm to carry out the examination as well as for preparing the final report.

The two refraction methods were compared using a power vector analysis method. Power vectors are a geometric representation of spherocylindrical refractive errors in 3 fundamental dioptric components: *M* (*M* = *S* + *C*/2), J0 (J0 = (−*C*/2) cos (2*α*) at an axis of *α* = 0 = 180°), and J45 (J45 = (−*C*/2) sin (2*α*) at an axis of *α* = 45°) [32, 33].

### 2.5. Statistical Analysis

Statistical analysis was performed with commercial software (SPSS v. 13.0; SPSS Inc., Chicago, IL). The normality of the refraction data was assessed using Shapiro–Wilk tests. We used paired and unpaired Student *t*‐test to compare the outcomes in this population. The Bland–Altman method was applied, and the 95% LoAs between the measurements obtained from *M*, J0, and J45 by the expert and those obtained by the SiviewExam® Expert system were evaluated for one randomly selected eye per participant [34, 35]. The clinically acceptable LoA was set at 0.7 D.

The intraclass correlation coefficients (ICCs) were calculated using a 1‐ or 2‐factor ANOVA for *M*, J0, and J45 for each pair of measurements [35, 36].

A calculated *p* value < 0.05 was considered statistically significant. Data are presented as the mean ± standard deviation.

## 3. Results

### 3.1. Demographics

A total of 107 (randomly selected) eyes of 107 patients (57% female and 43% male) were included in the study. The mean preoperative spherical equivalent (measured by the expert) was −2.10 ± 3.37 D (ranging from −11 D to +7.13 D), and the mean age was 35.7 ± 11.9 years (ranging from 19 to 69 years). Data are further detailed in Table 1, while Figure 2 describes the distribution in the *M* measurements across the population.

**TABLE 1:** Statistics of *M*, J0°, and J45° measurements by the expert.

Statistics – expert examination	*M* (D)	J0 (D)	J45 (D)
No. of patients	107	107	107
Mean ± SD (min/max)	−2.10 ± 3.37 (−11.0/7.13)	0.23 ± 0.53 (−1.13/1.95)	0.0 ± 0.36 (−2.50/1.13)

FIGURE 2(a) Scattergram and (b) box plot of *M* measurements by the expert and by SiviewExam expert system.

**Figure:**
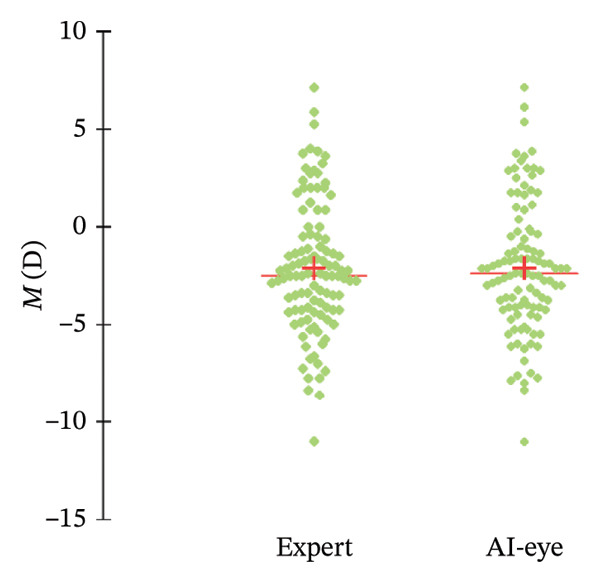
(a)

**Figure:**
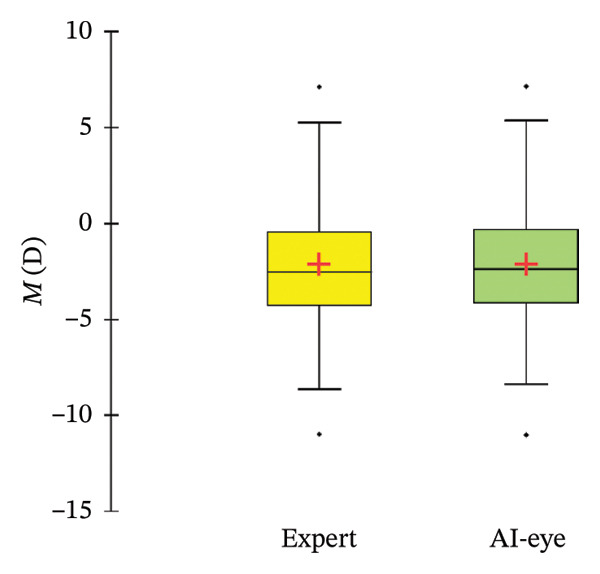
(b)

### 3.2. Comparison Between the Two Methods

The difference in the means was, respectively, 0.01 D (confidence interval [CI] 95%: −0.04 to +0.07 D), −0.05 D (CI 95%: −0.08 to −0.02 D), and 0.009 D (CI 95%: −0.01 to +0.03 D) at *M*, J0, and J45 (paired *t*‐test: *p* = 0.64, *p* = 0.002, and *p* = 0.43).

**FIGURE 3:**
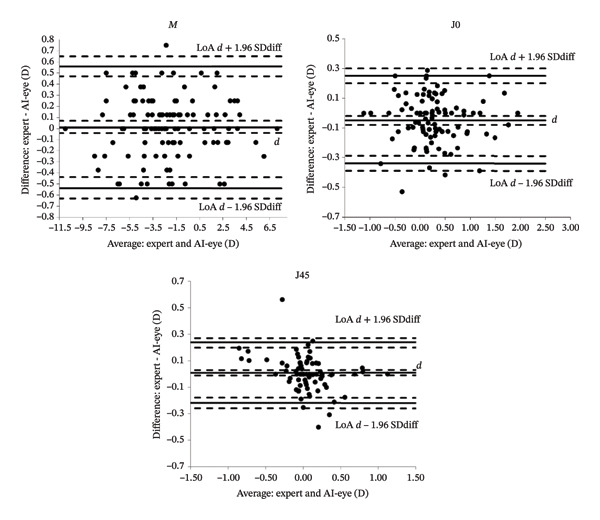
Bland–Altman plots comparing the limits of agreement of *M*, J0, and J45 between the measurement by an expert and the measurement by SiviewExam expert system. The central horizontal line indicates the mean of the differences *d*. The outer black lines indicate the limits of agreement. The black dashed lines delimit the CI 95% for *d* and the LoAs [37].

As shown in Figure 3, the LoAs for *M*, J0, and J45 were from −0.54 D (CI 95%: −0.63–−0.44) to 0.56 D (CI 95%: 0.47–0.65), from −0.34 D (CI 95%: −0.39–−0.29) to 0.25 D (CI 95%: 0.20–0.30), and from −0.22 D (CI 95%: −0.26–−0.18) to 0.24 D (CI 95%: 0.20–0.27), respectively.

As detailed in Table 2, the ICCs for the three components were *M* = 0.996, 0.996, and 0.996; J0 = 0.956, 0.957, and 0.960; and J45 = 0.951, 0.951, and 0.951. For J0, the *F*‐test and a consistency ICC higher than the absolute agreement ICC showed a bias.

**TABLE 2:** ICC values for expert and SiviewExam expert system measurements for *M*, J0, J45; *F* test to determine the bias between expert and SiviewExam expert system measurements of *M*, J0, and J45.

**ICC**	***M*** **95% CI** **^∗^** **σ**_**V**_ **/** **σ**_**R**_ **;** **σ**_**C**_ **/** **σ**_**R**_	**J0** **95% CI** **^∗^** **σ**_**V**_ **/** **σ**_**R**_ **;** **σ**_**C**_ **/** **σ**_**R**_	**J45** **95% CI** **^∗^** **σ**_**V**_ **/** **σ**_**R**_ **;** **σ**_**C**_ **/** **σ**_**R**_

ICC (1, 1): classic	0.996 0.995; 0.998 6.0%	0.956 0.938; 0.970 22.7%	0.951 0.929; 0.966 21.2%

ICC (2, 1): absolute agreement	0.996 0.995; 0.998 6.0%; 0.3%	0.957 0.933; 0.972 20.4%; 5.9%	0.951 0.929; 0.966 22.7%; 0.0%

ICC (3, 1): consistency	0.996 0.995; 0.998 6.0%; 0.3%	0.960 0.942; 0.973 20.4%; 5.9%	0.951 0.929; 0.966 22.7%; 0.0%

*F* test *F* = MSBM/MSE (mean square between measurements/mean square error)	*M* (SE) p 1.201 *p* = 0.28	J0 p 9.962 *p* = 0.002	J45 p 0.719 *p* = 0.40

^∗^ *σ*_*V*_ = standard deviation of the measurement, *σ*_*C*_ = standard deviation of the bias, *σ*_*R*_ = standard deviation of the measured quantity.

The ratio of the standard deviation of the measurement (*σ*_*V*_) to the standard deviation of the measured quantity (*σ*_*R*_) was 6.0%. The ratio of the standard deviation of the bias (*σ*_*C*_) to the standard deviation of the measured quantity (*σ*_*R*_) confirmed that the bias was zero for *M* and J45. For J0, the bias was 5.9% of the intersubject variability.

Finally, 100% of the reports produced by the SiviewExam® Expert system at the end of the examinations were accurate, with a 95% CI of 0 (0%: Wilson method).

## 4. Discussion

New automated subjective refraction systems must be evaluated against a gold standard, which is a subjective refraction performed manually by an experienced optometrist. This comparison ensures that the results obtained are clinically consistent.

To our knowledge, the results of this study are the most statistically robust in the literature to date, due to the use of a large sample of eyes.

We have demonstrated that there was no bias between the measurements of the spherical equivalent (*M*) and J45 and a clinically negligible bias (0.05 D) in the estimation of J0, with the SiviewExam® Expert system tending to give a slightly lower value than the expert. Moreover, the LoAs according to Bland–Altman for *M* (−0.54–+0.56), J0 (−0.34–+0.25), and J45 (−0.22–+0.24) were significantly lower than the expected interexaminer variability. In 2024, Carpena et al. assessed the interexaminer repeatability of subjective refraction across diverse age cohorts, an aspect not previously investigated. Based on their survey and previous research, they concluded to a 95% CI of repeatability of 0.75 D for *M* and between 0.25 D and 0.50 D for J0 and J45 [6, 16, 38, 39].

Also, there was a clinical agreement between the refraction measurements of the expert (gold standard) and SiviewExam® Expert system for *M*, J0, and J45, indicating that the SiviewExam® Expert system examination, as a diagnostic support system, can reasonably be considered a reliable aid and comparable to an expert’s examination.

The ratios of the standard deviation of the measurement (*σ*_*V*_) to the standard deviation of the measured quantity (*σ*_*R*_) confirmed that the ICCs were very good. For *M*, the ratio was 6.0%, which meant that the measurement error was only 6% of the intersubject variability. The ratios of the standard deviation of the bias (*σ*_*C*_) to the standard deviation of the measured quantity (*σ*_*R*_) confirmed that the bias was zero for *M* and J45. For J0, the bias was confirmed but negligible (only 5.9% of the intersubject variability) (Table 2).

It should also be noted that this study’s results were obtained from a population with a wide range of ages (19–69 years), ametropias (from −11 D to +7.13 D), and astigmatism (see Figure 2), which means they can be applied to healthy adults with any ametropia. However, further studies are needed to draw conclusions on the results concerning younger patients, those with KC, and the potential effects of cycloplegia.

Other systems for automated subjective refraction measurement have been studied. Bossie et al. [20] compared subjective refraction measurements between a standard digital refractor and the Chronos binocular refraction system, on 70 adults with normal vision and a corrected VA of +0.1 logMAR in each eye. The LoAs between the standard measurement and the tested system were similar to the SiviewExam® Expert system (−0.62; +0.68 for *M*, −0.24; +0.19 for J0, and −0.18; +0.16 for J45) but on a smaller and less diverse population, consisting only of myopes. Carracedo et al. [19] compared the Eye Refract binocular refraction system to traditional subjective refraction measurement on 99 subjects aged 7–69 years and showed less agreement between the two measurements than that observed with the SiviewExam® Expert system, with wider LoAs (close to ±1 for *M*, close to ±0.5 for J0, and between −0.3 and +0.2 for J45). Other studies by Carracedo et al. aimed at evaluating the Eye Refract system have shown an underestimation of hyperopia without the use of cycloplegic [21] and better results on healthy subjects compared with subjects with KC [23].

In a study published by Venkataraman et al. [24], semiautomated algorithms (integrated into the Vision‐R 800 Refractor) for subjective refraction measurement were compared with a standard measurement on 68 subjects aged 18–40 years. The results showed lower agreement between the measurements than those obtained in our study, with higher LoAs for the measurement of *M* (−0.8; +0.9 for the 1st algorithm and −1.0; +0.6 for the 2nd).

Moreover, the report provided by the SiviewExam® Expert system was found to be accurate according to an expert optometrist in 100% of the cases. This report is thus very useful to help optometrists in the management of the ocular correction of their patients by providing precise and time‐efficient assistance. It complements the refraction values by providing a targeted written analysis for each patient profile, assisting the expert in their diagnosis and the choice of the optimal prescription.

For example, one of the patients of the sample, a 26‐year‐old hyperopic adult who has never worn optical correction, was found +4.00 (−0.25) D on the right eye and +3.75 D on the left eye by the SiviewExam® Expert system. The supplementary analysis of the report advised not to fully correct the patient but proceed in stages, as the correction measured had a high probability of not being tolerated [13, 40].

The absolute search for time savings is not the priority for this algorithm, which aims more to achieve the optimization and customization of the correction while maintaining very good repeatability and reproducibility. A next step in the evaluation of the clinical performance of the SiviewExam® Expert system would likely be to study the subjective comfort of the patients.

The results of this study on a large adult population of over 100 patients with a wide range of ametropias showed that there was a clinical agreement in the results obtained with the SiviewExam® Expert system and by a professional expert. Although our findings are promising, they are restricted to a population aged 19–69 years without corneal pathologies or recent ocular surgeries. Additional research is required to determine how this approach performs in pediatric populations, individuals with complex refractive errors, and patients undergoing refractive surgery.

The integration of increasingly efficient algorithms into subjective refraction tools and the emergence of teleconsultation and tele‐expertise, as well as the progressive use of artificial intelligence, represent significant evolutions in the field of optometry and ophthalmology.

We can imagine that a future gradual integration of AI into subjective refraction systems like the SiviewExam® Expert system promises further improvements in terms of prescription personalization, accuracy, and timeliness of examinations and early detection of visual abnormalities. In addition, and very importantly, it should accelerate the spread of visual healthcare to a wider population that is currently under‐resourced. Indeed, the generalization and widespread use of such expert systems could represent a groundbreaking change in the quality of patient care (independent from operator) and access to healthcare in underserved areas. This would, in turn, foster equality of opportunities in accessing care and contribute to reducing health inequalities across societies worldwide.

Despite the potential benefits, proper clinician training and user education remain essential to ensure that automated refraction tools are used correctly and that their results are interpreted within the broader clinical context of each patient’s ocular health.

Initially, AI should improve the algorithms currently available in the expert systems such as the SiviewExam® Expert system by allowing machines to learn from the experience accumulated during millions of exams. Over time, these systems may pave the way for more personalized optical solutions by dynamically adjusting refractions based on individual patient profiles, visual habits, or even diurnal variations in refraction. Rigorous clinical evaluation and adequate training of professionals are, however, essential to maximize the benefits of these new technologies. Future evaluations should also address patient comfort and the user experience, as these factors are critical to ensuring accurate feedback during subjective refraction and successful integration into clinical workflow.

Overall, the progressive integration of AI into ophthalmic instruments offers a promising avenue to enhance diagnostic accuracy, improve repeatability of measurements, and ultimately augment patient satisfaction. Nonetheless, rigorous clinical validation and large‐scale, multicenter trials are necessary to confirm the generalizability of these early findings.

## Funding

This work was supported by SiView.

## Ethics Statement

The study and data acquisition were achieved with approval from the independent ethical review board South Mediterranean III (France, No. 2019.09.10_cinq_19.01641.190910 and from the Hopital Fondation Adolphe de Rothschild clinical research’s compliance department.

## Conflicts of Interest

Some of our co‐authors are consultants or have proprietary interest in SiView.

## Data Availability

The data that support the findings of this study are available on request from the corresponding author. The data are not publicly available due to privacy or ethical restrictions.
